# The hematobiochemical status of Wistar rat line under the bovine leukemia virus experimental infection

**DOI:** 10.14202/vetworld.2019.382-388

**Published:** 2019-03-12

**Authors:** Ekaterina Sergeevna Krasnikova, Fayssal Bouchemla, Alexander Vladimirovich Krasnikov, Roman Vladimirovich Radionov, Anastasia Sergeevna Belyakova

**Affiliations:** 1 Department of Microbiology, Biotechnology and Chemistry, Faculty of Veterinary Medicine, Vavilov Saratov State Agrarian University, Saratov, Russia; 2 Department of Animal Disease, Veterinarian and Sanitarian Expertise, Faculty of Veterinary Medicine, Vavilov Saratov State Agrarian University, Saratov, Russia

**Keywords:** cattle, danger to humans, hematobiochemical indicators, leukemia, Wistar rats

## Abstract

**Aim:**

This study aimed to elucidate the ability of the bovine leukemia virus (BLV) to integrate into cells of heterologous organisms, in particular, Wistar rats, and examine the manifestations of the pathological process that could be seen in them.

**Materials and Methods:**

Wistar rats - were divided into three groups. The first group (I) was fed milk of intact cows, the second (II) - milk of BLV-infected cows, and the third (III) - milk of cows, clinically BLV sick. Rats of all groups were divided into two subgroups: In the subgroup “a”, there were adult rats, and in the subgroup “b”, their offspring were included. At 3, 6, 9, and 12 months from the start of the experiment, the animals’ blood of each group was examined by polymerase chain reaction (PCR) and enzyme-linked immunosorbent assay for the presence of BLV provirus and specific anti-leukemia antibodies. A general and biochemical blood test was performed; pathological changes in the internal organs were recorded.

**Results:**

Using the PCR, the BLV infection was established in all experimental rats, whose immune response was expressed in varying degrees. At the initial stage of the infection, offspring rats were born healthy. The rats of the control groups Ia and Ib were intact to the BLV throughout the experiment. The biochemical blood tests have shown several signs of intoxication, endocrine disorders, and development of malignant processes in the experimental animals. There are also signs of liver, kidney, and myocardial damages, regardless of whether milk is infected or the cows are clinically leukemic. By the time, the experimental rats developed persistent thrombocytosis with an increase in the average volume of the blood platelets, which may be evidence of the leukemia infection by the megakaryocytic type. The most pronounced character of the change was in the offspring generation.

**Conclusion:**

Wistar rats can be considered as a suitable laboratory model to study the BLV pathogenesis. Rats are not BLV natural host, however, they developed the pathognomonic BL*V* infection symptoms when they were fed infected and leukemic cow’s milk.

## Introduction

Bovine leukemia virus (BLV), along with T-lymphotropic human I and II viruses, is oncogenic representatives of the genus *Deltaretrovirus* of the *Retroviridae* family. Currently, there are 10 genotypes and a large number of BLV subtypes common in all continents [1]. In the Russian Federation, since 1997, cattle leukosis has been ranked as the first disease among infectious diseases, with a trend of morbidity increase [2]. According to the official statistics, one-third of cattle in Russia are infected with BLV (1, 2, 4, 7, and 8 BLV genotypes were registered) [2-4]. It was established by Smirnov [5] that the BLV-1 genotype has the highest leukemogenicity and BLV-4 is the significantly less one.

The BLV affects a wide range of immune cells, which is, especially, actual, and it has been found in the epithelium of the mammary gland [6-10]. However, in another study, no correlation between breast cancer in women and the presence of BLV antibodies has not been revealed [11, 12]. Disputing this, it had been questioned if veterinary kits are more suitable for human studies [13]. The question of the potential danger of the BLV for humans is a new and highly topical subject of scientific controversy.

The presence of antibodies without studying the dynamics of their accumulation after a single administration of the antigen cannot be considered as indisputable evidence of the infectious process. These data indicate the immunoreactivity of the experimental animals toward the BLV and, obviously, the presence of this virus in the blood of a person with clinical manifestations of leukemia. It was revealed the fact that the BLV interspecies transmission to rabbits by alimentary tract, thereby confirming the infectious properties of milk from the BLV infected cows [14].

This study aimed to elucidate the ability of the bovine leukemia virus (BLV) to integrate into cells of heterologous organisms, in particular, Wistar rats, and examine the manifestations of the pathological process that could be seen in them.

## Materials and Methods

### Ethical approval

The study was conducted according to the guidelines laid down by the European Convention for the Protection of Vertebrates used for experimental and other scientific purposes and in accordance with the local laws and regulations (N 1/04.9.2018).

### Area and design of the study

The object of the study was laboratory rats of the Wistar line (n=60). The rats were divided into three equal groups at the rate of 2–3 females per 1 male and they were kept in identical conditions; a full-fledged ration and received a daily fresh raw cow milk. The first group (I) of rats was fed milk of intact cows, the second (II) - milk from BLV-infected cows, and the third (III) - milk from cows, clinically BLV ill (based on data from the state service). The obtained rats’ progeny was kept together with their parents. Rats in Groups I, II, and III were divided into two subgroups: In the subgroup “a”, there were adult rats, and in the subgroup “b”, their offsprings were included (from the second progeny and so on). At 3, 6, 9, and 12 months from the start of the experiment, the animals of each group were fulfilled a blood aspiration from the caudal lateral vein. In the hematological and molecular biological studies, tubes with a stabilizer K3 EDTA for biochemical and serological with a clot activator (PUTH, Russia) were used.

### Molecular identification of BLV isolates

Either presence or absence of the BLV provirus in the blood and milk sampling were established by the method of classical polymerase chain reaction (PCR), using the *LEIKOZ* set (InterLabService, Russia) and its own development–multiplex PCR (RF patent No. 2615465). The purpose was to exclure the concomitant leukemia infection of the bovine immunodeficiency virus [15]. Amplification and recording of the results were carried out on the equipment *T100* and *GelDoc XP PLUS* (Bio-Rad, USA). The detection of the specific antileukemia antibodies in the blood serum of rats was performed by the method of solid-phase enzyme-linked immunosorbent assay using the kit for BLV detecting antibodies in blood serum and milk by the enzyme immunoassay method (variant No. 1 - screening) produced by the Kursk Biofactory - firm *“BIOK”* (Russia) on the equipment Multiskan (“Thermo Scientific,” USA).

### General blood test (GBT)

A GBT was performed using the Abacus Junior 5 vet hematological automatic analyzers and original consumables (Diatron MI Zrt., Hungary). Biochemical studies were carried out using the *StatFax 3300* analyzer with reagents, manufactured by *AO (DIAKON-DS) (Russia)*.

### Statistical analysis

The results of the studies were processed using the statistics 6 programs (Borland Software Corporation, USA) based on a computer running Windows 7 and an Intel Core 2 Duo processor.

## Results and Discussion

Our research has shown that the white laboratory rats of the Wistar line are susceptible to the BLV oral infection: After 3 months of having milk from infected and leukemic cows, the rats were BLV infected. The exception was the Group IIb - monthly pups, which apparently were protected by colostral antibodies. In the Group IIIa, the animals had a lack in the immune response.

It is well known that, within the leukemic infection, there are defective lymphocytes and long-lived suppressors, and the serum of the blood of immune animals contains an antibody inhibitor, which explains the long latent period in the immune response [6,16]. Animals of Group IIIb were not examined because they were in the neonatal period. It should be mentioned that the consumption of milk from the BLV-infected and leukemic cows led to a violation in the reproductive function in rats, as their offspring began to appear much later than in rats that were fed milk from intact cows.

PCR was proceeded at 6, 9, and 12 months of the experiment, where the BLV infection was established in all experimental animals, except the rats of Groups I and Ib which were intact to the BLV throughout the experiment. The immune response was also in a varying degree expressed.

For the general blood analysis (Tables-1-4) and the results of the biochemical study of rat blood (Tables-5-8), we relied on to the reference values of Wistar rats, given in the reference book of the physiological, biochemical, and biometric indices of the experimental animal norm [17].

**Table-1:** Hematological parameters of rats (experiment of 3 months).

Indicator	Group of animals/age					

	Ia/9 months	Ib/2 months	IIa/9 months	IIb/1 month	IIIa/9 months	IIIb/neonatal
RBC, 10^12^/L	7.3±0.7	6.1±0.6	7.3±0.7^#^	5.9±0.5	5.7±0.5*^#^	–
HGB, g/l	139.5±14.0	125.3±1.1	139.1±12.6^#^	119.7±11.4*	115.3±10.9*^#^	–
MCHC, g/l	382.0±32.2	404.0±37.8	405.0±38.8	406.0±39.2	423.0±41.1	–
RDWс, %	14.3±1.3	13.7±1.2	14.1±1.3	17.9±1.6*	15.4±1.3	–
MCV, fl	47.8±4.4	50.5±4.9	48.2±4.6	51.3±4.8	45.4±4.2	–
WBC, 10^9^/L	11.5±1.1	11.7±1.1	21.2±1.9*^#^	14.6±1.3*	7.4±0.7*^#^	–
LYM, %	66.8±6.6	66.8±6.6	63.7±6.5^#^	57.6±5.6*	31.1±3.1*^#^	–
MID, %	6.1±0.6	6.0±0.6	8.1±0.8*	4.0±0.4*	7.7±0.7*	–
GRA, %	27.1±2.5	27.2±2.6	28.2±2.6^#^	38.4±3.3*	61.2±5.8*^#^	–
PLT, 10^9^/L	440.0±43.0	514.0±49.2	695.0±67.9*^#^	694.0±68.3*	754.0±72.2*^#^	–
MPV, fL	5.9±0.5	5.5±0.5	6.7±0.6*	7.6±0.7*	7.1±0.7*	–

* The difference between the test group and the control group;

# The difference between the experimental groups among themselves; p≤0.1

**Table-2:** Hematological parameters of rats (experiment of 6 months).

Indicator	Group of animals/age					

	Ia/12 months	Ib/5 months	IIa/12 months	IIb/4 months	IIIa/12 months	IIIb/3 months
RBC, 10^12^/L	7.9±0.7	7.6±0.7	7.0±0.7*	8.1±0.7	6.7±0.6*	6.9±0.6*
HGB, g/l	150.0±15.0	151.0±14.8	140.0±14.0	148.0±13.8	146.014.1	150.0±14.5
MCHC, g/l	384.0±30.9	396.0±31.1	318.0±29.4*	308.0±28.7*	341.0±32.2*	332.0±32.1*
RDWс, %	12.1±1.1	11.6±10.9	12.9±1.1	15.4±1.4*^#^	11.8±1.1	10.8±0.9^#^
MCV, fl	50.1±4.6	51.1±4.9	62.7±6.1*	58.7±5.6*^#^	63.5±6.2*	65.2±6.3*^#^
WBC, 10^9^/L	7.3±0.6	9.4±0.9	15.8±1.4*	15.5±1.5*^#^	14.0±1.4*	17.9±1.6*^#^
LYM, %	62.2±6.1	63.0±6.2	56.2±5.3^#^	66.3±6.1^#^	69.7±6.7*^#^	77.3±7.6*^#^
MID, %	4.3±0.4	4.4±0.4	3.9±0.3	4.1±0.4^#^	4.0±0.3	3.1±0.3*^#^
GRA, %	33.5±3.2	32.6±2.8	39.9±3.6*^#^	29.6±2.7^#^	26.3±2.5*^#^	19.6±1.7*^#^
PLT, 10^9^/L	505.0±48.6	508.0±47.5	647.0±63.6*	624.0±61.6*	697.0±68.4*	663.0±65.5*
MPV, fL	5.4±0.4	6.0±0.6	6.8±0.6*	6.6±0.6*^#^	7.4±0.7*	8.2±0.7*^#^

* The difference between the test group and the control group;

# The difference between the experimental groups among themselves; p≤0.1

**Table-3:** Hematological parameters of rats (experiment of 9 months).

Indicator	Group of animals/age					

	Ia/15 months	Ib/8 months	IIa/15 months	IIb/7 months	IIIa/15 months	IIIb/6 months
RBC, 10^12^/L	7.3±0.6	7.8±0.7	5.9±0.6*	5.1±0.5*	5.6±0.5*	4.7±0.4*
HGB, g/l	141.0±13.8	146.0±14.1	124±12.2*	108.0±9.8*	128.0±12.4*	100.0±9.7*
MCHC, g/l	394.0±33.5	399.0±34.1	328.0±31.6*	308.0±29.4*	350.0±33.7*	315.0±30.8*
RDWс, %	11.6±1.1	11.5±1.1	12.2±1.1	17.2±1.3*	13.0±1.2*	14.0±1.2*
MCV, fl	52.1±5.1	53.4±5.0	63.9±6.1*	68.8±6.9*	65.0±6.3*	66.8±6.3*
WBC, 10^9^/L	9.4±0.8	10.4±0.9	14.3±1.2*^#^	15.4±1.5*^#^	17.6±1.6*^#^	20.4±1.8*^#^
LYM, %	54.0±5.2	56.1±5.3	57.0±5.4	57.6±5.5*^#^	61.7±5.9*	68.9±7.0*^#^
MID, %	6.5±0.6	4.9±0.5	5.6±0.5*	6.7±0.5*	5.9±0.6*	6.7±0.6*
GRA, %	39.5±3.7	39.0±3.8	37.4±3.6^#^	35.7±3.3^#^	32.4±3.1*^#^	24.4±2.3*^#^
PLT, 10^9^/L	530.0±52.4	491.0±45.1	668.0±64.2*^#^	650.0±63.4*^#^	684.0±66.3*^#^	890.0±90.0*^#^
MPV, fL	6.1±0.6	6.2±0.6	7.0±0.7*^#^	6.9±0.7*^#^	6.9±0.6*^#^	8.5±0.7*^#^

* The difference between the test group and the control group;

# The difference between the experimental groups among themselves; p≤0.1

**Table-4:** Hematological parameters of rats (experiment of 12 months).

Indicator	Group of animals/age					

	Ia/18 months	Ib/11 months	IIa/18 months	IIb/10 months	IIIa/18 months	IIIb/9 months
RBC, 10^12^/L	7.9±0.7	7.8±0.7	7.9±0.8	7.7±0.6^#^	7.8±0.7	8.0±0.8
HGB, g/l	145.0±13.8	135.0±12.7	128.0±11.6*	114.0±10.9*	125.0±11.8*	116.0±10.1*
MCHC, g/l	393.0±35.8	401.0±38.7	253.0±24.6*	266.0±25.5*	258.0±25.6*	277.0±25.9*
RDWс, %	10.8±0.9	11.4±1.1	18.2±1.6*	21.3±1.9*^#^	17.4±1.6*	15.9±1.4*^#^
MCV, fl	49.0±4.6	53.0±5.1	64.0±6.2*	62.0±6.1*	62.0±5.8*	60.0±5.4*
WBC, 10^9^/L	8.7±0.8	9.2±0.8	15.9±1.4*	23.4±2.2*^#^	14.9±1.3*	19.8±1.7*^#^
LYM, %	50.8±4.6	56.6±5.2	57.0±5.3*	34.7±3.2	56.6±5.2*	69.2±7.0
MID, %	4.7±0.4	5.2±0.5	6.7±0.6*^#^	4.3±0.4*^#^	3.7±0.3*^#^	11.3±1.2*^#^
GRA, %	44.5±4.2	38.2±3.6	36.3±3.3*	61.1±5.9*^#^	39.7±3.7*	19.5±12.0*^#^
PLT, 10^9^/L	472.0±45.2	357.0±33.7	1343.0±132.0*^#^	1833.0±181.0*	2057.0±201.0*^#^	1685.0±165.0*
MPV, fL	5.8±0.5	6.1±0.6	8.3±0.8*^#^	9.5±0.9*	9.5±0.9*^#^	8.8±0.8*

* The difference between the test group and the control group;

# The difference between the experimental groups among themselves; p≤0.1

The data in Table-1 indicate a thrombocytosis in the animals of the experimental groups, while the average volume of platelets slightly increases. In the IIa and IIIa, the development of an allergy could be assumed because the average blood cells have been slightly exceeded. In Group IIIa, the increased number of neutrophils indicates an active response at the cellular level. In Group IIa, a leukocytosis was mentioned while the overall ratio of leukocyte fraction still maintained.

Following the data given in Table-2 which were taken 6 months after the start of the experiment, Groups IIIa and IIIb exhibited signs of an hemolytic anemia, such as a decrease in the number of erythrocytes against the background of the normal hemoglobin levels and, on the other hand, an increase in the average volume of red blood cells, which could be a result of an intoxication. In addition, in these groups, we have a slight lymphocytic leukocytosis. Thrombocytosis was again noticed in animals of the experimental groups with an increase in the average volume of thrombocytes, which may be evidence of a hematopoiesis violation and, in particular, cell maturation. Recent studies have shown that people with acute leukemia often develop primary thrombocytosis, due to a violation of bone-marrow hematopoiesis [18].

The third analysis of the rats’ hematological parameters (Table-3) revealed a tendency for the continuous development of emerging processes. In Group IIb, the indicator of the width of the erythrocyte distribution in the volume again was slightly higher, which might indicate the development of an anisocytosis in animals of this group. The tendency of decreasing in the quantities of erythrocytes and hemoglobin in rats of experimental groups was mentioned, which could be a sign of a disorder. In Group IIIb, signs of lymphocytic leukemia increased. The quantity and size indicators of thrombocytes in the experimental animals were also exceeded.

The final analysis of the rats’ hematological parameters (Table-4) shows that, when the number of erythrocytes in groups of experimental animals is restored, the hemoglobin of the blood and the amount of hemoglobin per erythrocyte are reduced, and at the same time, the cell volume is exceeded and the exponent of erythrocytes’ distribution width is increased with the volume. All this could be a consequence of hemolysis of red blood cells due to the intoxication, which led to the presence of a large number of young forms of erythrocytes in the blood. In the Group IIIb, there are insignificant signs of lymphocytic leukemia and, in the Group IIb, leukocytosis of neutrophil type. In all experimental groups, there was a significant increase in the number of thrombocytes accompanied by an increase in the average cell volume.

The obtained data correlate to the opinion of several authors. Thus, Donnik *et al*. [19] revealed pathological forms of blood cells, in particular, erythrocytes, in BLV-infected animals. Furthermore, it was established a wave-like arrhythmic dynamics of hematological indicators of leukemic animals, which defined as a process cyclicity. Each cycle includes the phases of remission and exacerbation (temporary reduction and increase in leukocytosis) in varying degrees of severity. When the leukemic process develops to the tumor stage, the cyclicity of changes in hematological parameters becomes more pronounced, and the amplitude of the oscillations significantly increases [20]. A similar phenomenon of cyclicity was observed in our experiment; moreover, cyclicity was characteristic, not only of white but also of red blood cells. A steady positive dynamics were observed in blood platelets. The revealed changes of thrombocyte indicators might also be an indication of immunosuppression, as it was revealed too, by Rozhkov *et al*. [21], that thrombocytes have recently been assigned a significant role in the formation of immunity. At the meantime, Pavlova *et al*. [22] have concluded that the change in the quantitative composition of immunocompetent blood cells within leukemia is not a violation of cellular homeostasis but serves as a marker for the structural response of the body to maintaining its functional homeostasis.

As follows from the data, presented in Table-5, in the Group IIIa, there is an increase in creatinine, which probably indicates a renal pathology. In the Group IIb, the increase in the total protein is due to the globulin fraction, which indicates a high immune status of animals. In the Groups IIa and IIIa, the Rytis coefficient was 4 and 3, respectively, which might indicate a cardiac pathology.

**Table-5:** Biochemical indices of rat blood (experiment of 3 months).

Indicator	Group of animals/age					

	Ia/9 months	Ib/2 months	IIa/9 months	IIb/1 months	IIIa/9 months	IIIb/neonatal
Alkaline phosphatase, U/L	261.9±25.3	697.5±68.7	195.9±18.8*^#^	579.5±55.3*	265.5±26.1^#^	–
Urea, mmol/L	7.6±0.7	6.6±0.6	8.0±0.8^#^	6.9±0.6	6.4±0.6*^#^	–
Creatinine, μmol/L	51.5±4.8	44.6±4.1	39.2±3.6*^#^	52.5±5.1*	83.0±8.2*^#^	–
Glucose, mmol/L	24.7±2.2	17.2±1.6	12.1±1.1*^#^	14.8±1.4*	14.4±1.3*^#^	–
Total protein, g/l	74.5±7.1	92.7±8.9	64.2±6.2*	80.1±7.8*	66.3±6.5*	–
Albumin, g/l	38.1±3.6	48.8±4.4	39.7±3.7	33.5±3.1*	38.8±3.6	–
ALT, E/L	108.6±10.1	81.3±8.1	93.3±9.2*^#^	62.7±6.2*	42.5±4.1*^#^	–
AST, E/L	176.5±17.5	162.5±16.1	381.3±37.8*^#^	123.3±11.9*	118.7±11.6*^#^	–
Bilirubin total, μmol/L	3.5±0.3	3.1±0.3	3.3±0.3	12.1±1.1*	3.2±0.3	–

* The difference between the test group and the control group;

# The difference between the experimental groups among themselves; p≤0.1

Throughout 6 months of the experiment, changes in the biochemical parameters of rat blood relative to the control group became more pronounced (Table-6). In the Group IIa, there is a significant decrease in the level of the enzyme alkaline phosphatase, which indicates some disorders in the thyroid gland, primarily its hyperfunction. In the Groups IIIa and IIIb, the globulin fraction increases, which could be a consequence of the progression of the infectious process.

**Table-6:** Biochemical indices of rat blood (experiment of 6 months).

Indicator	Group of animals/age					

	Ia/12 months	Ib/5 months	IIa/12 months	IIb/4 months	IIIa/12 months	IIIb/3 months
Alkaline phosphatase, U/L	287.3±25.9	226.5±21.9	150.3±14.5*^#^	723.6±70.9*	251.2±24.7*^#^	308.4±28.5*
Urea, mmol/l	6.7±0.6	7.5±0.7	5.1±0.5*^#^	4.6±0.4*	3.6±0.3*^#^	4.8±0.4*
Creatinine, μmol/L	59.3±5.6	56.8±5.3	48.4±4.4*^#^	73.3±6.9*^#^	62.1±6.1^#^	69.9±6.8*^#^
Glucose, mmol/l	3.5±0.3	4.31±0.4	7.4±0.7*^#^	7.5±0.7*^#^	6.3±0.6*^#^	12.9±1.1*^#^
Total protein, g/l	63.3±6.1	69.1±6.5	74.2±7.1*^#^	61.1±5.9	66.1±6.4^#^	71.6±6.9*^#^
Albumin, g/l	31.8±2.9	30.1±2.8	39.7±3.7*^#^	30.1±2.9^#^	26.2±2.3*^#^	24.8±2.1*^#^
ALT, E/L	52.8±5.1	69.8±6.5	44.7±4.2*	70.2±6.7^#^	42.4±4.1*	80.8±7.8*^#^
AST, E/L	112.9±11.6	199.4±18.9	222.8±21.3*^#^	234.0±22.4*	178.6±16.9*^#^	237.2±22.7*
Bilirubin total, μmol/l	2.8±0.3	1.2±0.1	7.6±0.6*^#^	15.1±1.4*^#^	6.4±0.4*^#^	12.8±1.2*^#^

* The difference between the test group and the control group;

# The difference between the experimental groups among themselves; p≤0.1

Rytis coefficient in the experimental groups continued to grow and testify about cardiac pathology. The increase in bilirubin, as well as the increase in the hepatic enzyme alanine aminotransferase (ALT) in the Group IIIb, may be a marker of the beginning liver pathology or hemolytic anemia in the experimental groups. A high level of alkaline phosphatase against the background of an increase in the amount of bilirubin and limiting values of ALT in the Group IIb, as well as in the offspring in Group III, ought to be markers of hepatobiliary system diseases. Increased creatinine in the blood serum of Group IIb and IIIb rats gave a sign of beginning renal failure because an increase in creatinine concentration in renal failure occurs earlier than an increase in urea concentration.

After another 9 months from the beginning of the experiment, we observed a further decrease in the level of the enzyme alkaline phosphatase, which may indicate the development of endocrine disorders in rats of experimental groups. The most striking changes were observed in the offspring of Group III, having milk from sick cows:

- High creatinine and low total protein in the complex indicate the predominance of catabolic processes in the body over anabolic;
- Low glucose against a high-calorie diet - an indicator of the state of intoxication;
- The globulin fraction of blood proteins is not significantly represented - pronounced immunosuppression;
- A consistently low level of ALT, a measure of malignant neoplasms in the body;
- High *Rytis coefficient* - severe myocardial damage;
- High bilirubin - liver damage and hemolytic processes.

In other experimental groups, biochemical changes may be signs of kidney damage (increase in urea and creatinine), liver (synchronous growth of bilirubin and ALT), myocardium (high Rytis coefficient), and a marked immune response in the Group IIb (increase in the globulin fraction of protein).

The final study of blood serum (Table-8) shows the most significant changes in biochemical indices in the group of animals IIIb - it is possible to assume violations in the liver, kidneys, heart, hormonal background, and development of oncological diseases. In a recent study, it was found that men with acute leukemia have dysfunction of the endocrine system of the pituitary gland, which leads to a metabolic disorder [23]. Alignment of the level of alkaline phosphatase in the blood serum in the experimental groups may be a consequence of its increase due to the hepatic fraction since the further growth of ALT and bilirubin is noted. All these indicate the destruction of hepatocytes and a decrease in the function of the organ. The increased indices of urea and creatinine are most likely a consequence of renal failure. It should be noted a decrease in the immune response in animals of all experimental groups (low globulin). A high level of glucose can be an indicator of dysfunction of metabolic processes, as a result of hormonal imbalance.

**Table-7:** Biochemical indices of rat blood (experiment of 9 months).

Indicator	Group of animals/age					

	Ia/15 months	Ib/8 months	IIa/15 months	IIb/7 months	IIIa/15 months	IIIb/6 months
Alkaline phosphatase, U/L	224.3±21.2	334.3±32.4	203.4±19.7*^#^	122±12.1*^#^	144.5±13.7*^#^	190.2±18.5*^#^
Urea, mmol/L	4.8±0.3	7.2±0.7	6.7±0.6*^#^	7.4±0.7	3.5±0.3*^#^	6.7±0.6
Creatinine, μmol/L	52.1±5.9	54.4±5.3	64.4±6.2*	69.5±6.7*^#^	60.1±5.8*	75.2±7.2*^#^
Glucose, mmol/L	5.2±0.5	7.5±0.7	5.4±0.5	8.9±0.8*^#^	5.8±0.5*	2.1±0.2*^#^
Total protein, g/L	47.2±4.5	44.3±4.2	45.5±4.4	72.1±7.1*^#^	50.8±4.7	40.5±3.8^#^
Albumin, g/L	28.5±2.6	29.6±2.7	30.6±2.9	29.2±2.8^#^	28.2±2.7	32.9±3.1*^#^
ALT, E/L	52.8±5.1	65.2±6.4	62.1±5.8*^#^	50.1±4.7*^#^	42.4±4.1*^#^	25.9±2.2*^#^
AST, E/L	130.2±11.5	200.5±18.9	232.1±23.1*^#^	232.7±22.1*	174.3±16.9*^#^	213.3±20.8*
Bilirubin total, μmol/L	2.8±0.2	4.4±0.4	11.9±1.1*^#^	6.3±0.6*^#^	7.4±0.7*^#^	12.8±1.1*^#^

* The difference between the test group and the control group;

# The difference between the experimental groups among themselves; p≤0.1

**Table-8:** Biochemical indices of rat blood (experiment of 12 months).

Indicator	Group of animals/age					

	Ia/18 months	Ib/11 months	IIa/18 months	IIb/10 months	IIIa/18 months	IIIb/9 months
Alkaline phosphatase, U/L	370.6±36.6	337.3±51.3	298.9±28.6*^#^	216.1±20.9*^#^	461.3±44.8*^#^	295.2±27.9*^#^
Urea, mmol/L	6.4±0.6	5.5±0.5	8.0±0.8*^#^	10.8±1.0*^#^	9.2±0.9*^#^	9.3±0.9*^#^
Creatinine, μmol/L	50.8±4.8	66.2±6.4	106.7±10.1*	76.4±7.4*	105.6±10.2*	74.4±7.3*
Glucose, mmol/L	4.6±0.4	5.2±0.5	6.2±0.6*^#^	14.7±1.5*^#^	24.9±2.2*^#^	12.6±1.2*^#^
Total protein, g/L	72.0±7.1	62.9±6.1	46.7±4.3*^#^	54.2±5.1*^#^	67.8±6.6^#^	62.5±5.9^#^
Albumin, g/L	38.8±3.7	37.8±3.6	37.8±3.7	37.9±3.5	44.1±4.2*	41.6±3.9
ALT, E/L	58.8±5.7	79.7±7,7	90.3±8.9*	94.5±8.1*^#^	99.4±9.8*	46.7±4.4*^#^
AST, E/L	124.4±12.8	172.3±16,2	272.0±26.1*	277.6±25.9*	268.4±23.6*	233.7±22.2*
Bilirubin total, μmol/L	4.4±0.4	4.6±0.4	14.1±1.4*	12.2±1.2*^#^	15.1±0.5*	15.8±0.5*^#^

* The difference between the test group and the control group;

# The difference between the experimental groups among themselves; p≤0.1

Thus, the rates of alkaline phosphatase were the most dynamic in the rat serum. The indices of urea and creatinine in experimental animals increase by time, the same as the blood glucose. In general, the indicators of total protein and albumin were relatively stable. The enzymes such as AST and ALT had small positive dynamics, while the total blood bilirubin was greatly increased in the experimental rats by contribution to the control animals and had a pronounced positive dynamic over time.

## Conclusion

The thrombocytosis was also mentioned in the BLV-infected cows, whose milk had been given rats, and calves were obtained from these cows. In animal farms, where the experiment was conducted, the clinical form of leukemia was not often recorded, but it happens that animals, in whose leukogram of lymphocytes’ level is normal, perish from the phenomena of proliferative processes.

Proliferative processes were constantly recorded in animals of the Group IIIb, whose immune response was expressed to the least extent compared with other experimental rats. However, weak antigenic reactions stimulate the growth of highly antigenic tumors, so the frequency of neoplasia in infected patients with cellular immunodeficiency is a thousand times higher than the average in the population [24].

The consequence of which might be a circulating type of virus in this farm that provokes the development of lymphocyte leukemia to a lesser extent than local proliferative processes and megakaryocytic leukemia. Moreover, what is especially important, changes in the cattle body are similar to changes in rats, which are not natural hosts of the BLV.

## Authors’ Contributions

ESK designed the work. All authors conducted the research work. Data analysis and manuscript drafted by AVK, RVR, and ASB under the guidance of FB and ESK. All authors read and approved the final manuscript.
